# Profiling of serum factors associated with *Staphylococcus aureus* skin and soft tissue infections as a foundation for biomarker identification

**DOI:** 10.3389/fimmu.2023.1286618

**Published:** 2023-11-20

**Authors:** Elke S. Bergmann-Leitner, Eugene V. Millar, Elizabeth H. Duncan, David R. Tribble, Patrick M. Carey, Michael W. Ellis, Katrin Mende, Jason W. Bennett, Sidhartha Chaudhury

**Affiliations:** ^1^ Immunology Core, Biologics Research and Development, Walter Reed Army Institute of Research, Silver Spring, MD, United States; ^2^ Infectious Disease Clinical Research Program, Department of Preventive Medicine and Biostatistics, Uniformed Services University of the Health Sciences, Bethesda, MD, United States; ^3^ Henry M. Jackson Foundation for the Advancement of Military Medicine, Inc., Bethesda, MD, United States; ^4^ Martin Army Community Hospital, Fort Moore, GA, United States; ^5^ Department of Medicine, University of Toledo College of Medicine and Life Sciences, Toledo, OH, United States; ^6^ Brooke Army Medical Center, Joint Base San Antonio, Fort Sam Houston, TX, United States; ^7^ Multidrug-Resistant Organism Repository and Surveillance Network, Walter Reed Army Institute of Research, Silver Spring, MD, United States; ^8^ Center Enabling Capabilities, Walter Reed Army Institute of Research, Silver Spring, MD, United States

**Keywords:** *Staphylococcus aureus*, methicillin-resistant *Staphylococcus aureus* (MRSA), Skin and soft tissue infection, immunology, cytokine, biomarker

## Abstract

**Background:**

People living in close quarters, such as military trainees, are at increased risk for skin and soft tissue infections (SSTI), especially those caused by methicillin-resistant *Staphylococcus aureus* (MRSA). The serum immune factors associated with the onset of SSTI are not well understood.

**Methods:**

We conducted a longitudinal study of SSTIs, enrolling US Army trainees before starting military training and following up for 14 weeks. Samples were collected on Day 0, 56, and 90. Serum chemokines and cytokines among 16 SSTI cases and 51 healthy controls were evaluated using an electro-chemiluminescence based multiplex assay platform.

**Results:**

Of 54 tested cytokines, 12 were significantly higher among SSTI cases as compared to controls. Among the cases, there were correlations between factors associated with vascular injury (i.e., VCAM-1, ICAM-1, and Flt1), the angiogenetic factor VEGF, and IL-10. Unsupervised machine learning (Principal Component Analysis) revealed that IL10, IL17A, C-reactive protein, ICAM1, VCAM1, SAA, Flt1, and VGEF were indicative of SSTI.

**Conclusion:**

The study demonstrates the power of immunoprofiling for identifying factors predictive of pre-illness state of SSTI thereby identifying early stages of an infection and individuals susceptible to SSTI.

## 1 Introduction

Infections caused by *Staphylococcus aureus*, particularly methicillin-resistant *S. aureus* (MRSA), are common and associated with substantial healthcare-associated costs (1–3). The severity of *S. aureus* caused skin and soft tissue infections (SSTIs) can range from mild to life-threatening and recurrence after initial infections have been reported (4). While up to 30% of individuals are colonized, the underlying conditions governing colonization or resistance to colonization are still unknown (5). Vaccination remains an ideal, but elusive prevention strategy, as no *S. aureus* vaccine has shown protective efficacy in humans (6, 7). Progress in this domain has been impeded by an incomplete understanding of the pathogenesis of *S. aureus* infection, as well as the inability to define immune correlates, whether antibody- or cell-mediated, that confer protection against the disease (8).

In the community setting, SSTI is the most common manifestation of *S. aureus* infection. People living in close quarters or with close skin-to-skin contact, such as military trainees are at increased risk for SSTI (2, 9) and effective disease prevention strategies in military settings have yet to be identified (7, 10–12). We conducted an observational, longitudinal study of SSTIs among US Army Infantry trainees at Fort Benning, GA (name changed to Fort Moore in 2023) to describe the epidemiology, natural history, and immunology of S*. aureus* colonization and infection in this high-risk population. Among a subset of participants with and without SSTI, we profiled serum cytokine responses to identify potential biomarkers associated with *S. aureus* colonization and/or SSTI. Computational data integration and multivariate analyses revealed factors with the potential to serve as biomarkers of early SSTIs.

## 2 Materials and methods

### 2.1 Study design and population

A longitudinal cohort study of SSTIs among US Army Infantry trainees at Fort Benning, GA, was conducted from 2015-2016. A description of the study design, setting, and population was published previously (13). The Institutional Review Board of the Uniformed Services University of the Health Science (Bethesda, MD) approved this study.

### 2.2 Case definition

A case of SSTI was defined as a trainee who presented to the Fort Benning outpatient Troop Medical Clinic (TMC) or was admitted to the Fort Benning Martin Army Community Hospital with a first-episode (during training), culture-confirmed staphylococcal SSTI (purulent cellulitis, abscess, paronychia, or infected blister). Individuals who presented to the TMC and were diagnosed with a culture-positive *S. aureus* SSTI were promptly prescribed antibiotics according to the clinical practice guidelines and were requested to return to the clinic for follow-up. Clinical cultures were obtained at the healthcare provider’s discretion as part of routine SSTI management.

### 2.3 Specimen collection

Sera were drawn from all study participants at three time points: [1] at baseline (enrollment); [2] on day 56 post-enrollment; and [3] on day 90 post-enrollment.

### 2.4 Subject selection

For the current study of serum biomarkers, a convenience sample of participants (n=67), including 16 participants with and 51 without SSTIs, were selected for inclusion. Since SSTI cases could present at any time during the observation period and blood draws for the cytokine analysis were only available at Day 0, Day 56, and Day 90, we defined pre-illness cases (n=16) as samples collected within seven days before the blood draw and only that time point was used for analysis. Control samples were defined as samples from subjects who did not have a SSTI during the study period. For these participants, serum from the baseline time point only was used for the analysis.

### 2.5 Cytokine measurement

To enable an in-depth profiling of serum cytokines/factors, the multiplex testing platform (Mesoscale Diagnostics (MSD), Gaithersburg, MD) was used for quantifying the serum cytokine concentrations of 54 factors. Each well contains up to ten individual spots for the quantitative detection of specific serum factors. The complete test panel consisted of: IFN-γ, bFGF, C-reactive protein, Eotaxin, Eotaxin-3, Flt-1, GM-CSF, ICAM-1, IL-10, IL-12/IL-23, IL-12p70, IL-13, IL-15, IL-16, IL-17A, IL-17AGenB, IL-17A/F, IL-17B, IL-17C, IL-17D, IL-1RA, IL-1 α, IL-1β, IL-2, IL-21, IL-22, IL-23, IL-27, IL-3, IL-31, IL-4, IL-5, IL-6, IL-7, IL-8, IL-8(HA), IL-9, IP-10, MCP-1, MCP-4, MDC, MIP-1α, MIP-1β, MIP-3α, PIGF, SAA, TARC, Tie-2, TNF-α, TNF-β, TSLP, VCAM-1, VEGF, VEGF-C, and VEGF-D. The quantification of serum factors was performed according to the manufacturer’s protocol. Briefly, sera were diluted in MSD diluent 1:2 and then added to V-plex plates. After 2 hrs incubation at RT, plates were washed with MSD wash buffer, and Sulfotag-conjugated detection antibodies were added. Finally, plates were washed with MSD wash buffer, MSD substrate buffer was added and plates were read using a QuickPlex SQ120. Data were expressed as pg/ml based on recombinant cytokine standard curves (14, 15). Initial data analysis including the determination of factor concentrations was done with the MSD Workbench™ software (Meso Scale Discovery, Rockville, MD).

### 2.6 Statistical analysis

Univariate analysis comparisons between groups [cases, controls (defined as participants without SSTI throughout study)] were made using a Shapiro-Wilk Normality Test followed by a student’s t test or a Wilcoxon signed rank test. Principal Component Analysis (PCA) was carried out by normalizing and scaling the log-transformed values. Data points were colored by group, and ellipses were generated corresponding to 50% confidence intervals for each group, to identify general trends in the data set. Correlation network plots were generated using pairwise Pearson correlation coefficients calculated from the log-transformed data. All statistical analyses were carried out in R using the *stats*, *ggplot2*, *corrplot, and corrr* packages.

## 3 Results

### 3.1 Baseline participant characteristics

**Figure 1:**
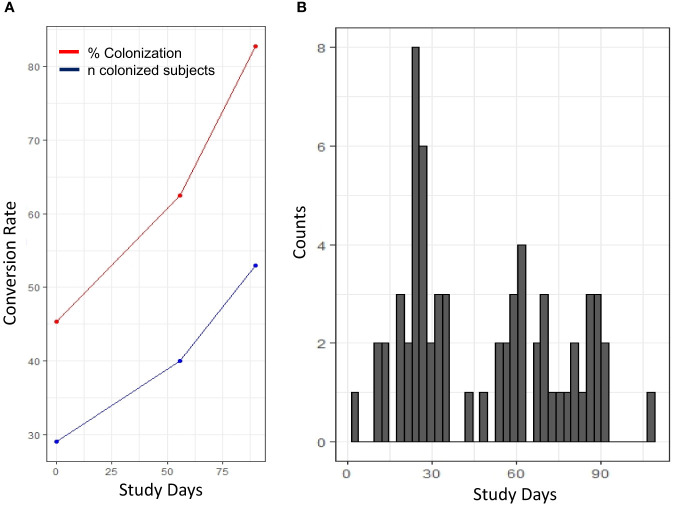
Longitudinal distribution of S. aureus colonization and occurrence of SSTI. **(A)** Changes in the frequency (% colonization, red) and number of colonized individuals (blue) shown in the course of the 90 study days. **(B)** Occurrence of SSTI diagnosed cases throughout the study. Histogram depicting the number of infected individuals at specific study day.

Serum samples from study participants were analyzed using a multiplex, electro-luminescence-based assay platform. The occurrence of SSTIs in the study population showed a bimodal distribution (**Figure 1**). For the purpose of identifying serum factors associated with a pre-illness state, only samples from individuals with SSTIs within 7 days before blood draw were utilized for the identification of candidate biomarkers. Since blood draws were not available for profiling between days 0 and 56, cases included in this analysis were from the later portion of the training (**Table 1**). The characteristics of the study participants (cases and controls) selected for establishing the serum cytokine profiling are summarized in **Table 1**. Statistical differences comparing cases with controls were observed in the age distribution and the use of antibiotics within 6 months prior to arriving at Fort Benning.

**Table 1:** Characteristics of study participants included in the serum cytokine analysis.

	Case (N=16)	Control (N=51)	Overall (N=67)	p-value
Age				
Median [Min, Max]	18.0 [17.0, 20.0]	19.0 [17.0, 29.0]	18.0 [17.0, 29.0]	
Sex				
Male	16 (100%)	51 (100%)	67 (100%)	
Race/Ethnicity				
White	14 (87.5%)	40 (78.4%)	54 (80.6%)	1
Black	0 (0%)	1 (2.0%)	1 (1.5%)	
Asian	0 (0%)	2 (3.9%)	2 (3.0%)	
Mixed/Other	2 (12.5%)	8 (15.7%)	10 (14.9%)	
History of SSTI prior to arrival				
Yes	1 (6.3%)	2 (3.9%)	3 (4.5%)	0.565
No	15 (93.8%)	49 (96.1%)	64 (95.5%)	
Antibiotic use in the 6 months prior to arrival				
Yes	3 (18.8%)	1 (2.0%)	4 (6.0%)	0.0396
No	13 (81.3%)	50 (98.0%)	63 (94.0%)	
Number of weeks in training at time of first infection				
Mean (SD)	11.3 (3.76)	NA (NA)	11.3 (3.76)	
Median [Min, Max]	13.4 [0.286, 14.1]	NA [NA, NA]	13.4 [0.286, 14.1]	

### 3.2 Detection and quantification of serum cytokines/chemokines

Statistical differences between SSTI cases (pre-illness, i.e., ≤ 7 days before SSTI diagnosis) and control subjects (baseline blood draw of individuals with no reported SSTI throughout the study) showed 15 of 54 serum factors significantly different at a 90% confidence level. Twelve of these factors were significant at p<0.05 (**Table 2**). Factors were classified based on their biological functions (i.e., chemokines, pro-inflammatory and Th-17 cytokines). Sera from SSTI cases showed significantly lower levels of chemokines Eotaxin, IL8, and MIP1β, pro-inflammatory cytokines (GM-CSF, IL2), Th17 cytokine IL17B, and the angiogenesis markers (Flt-1). In contrast, pro-inflammatory IL10, chemokines (MIP1α, IL16, Eotaxin3). Th17 cytokine IL17A, Vascular injury factors (CRP, ICAM1, VCAM1, SAA), and the angiogenesis factor VEGF, were significantly increased compared to control participants.

**Table 2:** Comparison of measured serum factors that differed between participants with versus without *S. aureus* SSTI.

Classification^1^	Factor	Median (*IQR)* ^1^		p-value^2^
		Case n=16	Control n=51	Case vs Control
Pro-/anti-inflammatory	IFNγ	3.0 *(2, 7)*	2.18 *(1.5, 3.2)*	0.068
	GM-CSF	0.001 *(0, 0.07)*	0.07 *(0, 0.12)*	0.03^*^
	IL10	0.35 *(0.29, 0.68)*	0.29 *(0.21, 0.42)*	0.03^*^
	IL2	0.11 *(0.07, 0.22)*	0.15 *(0.09, 0.18)*	0.7
Chemokine	Eotaxin	213 *(19, 338)*	269 *(216, 372)*	0.087
	Eotaxin3	21 *(17, 25)*	20 *(15, 29)*	0.05^*^
	IL16	244 *(204, 271)*	208 *(167, 246)*	0.074
	IL8	0 *(0,0)*	2 (*0, 67*)	0.006^*^
	MCP4	88 *(72, 150)*	115 *(90, 115)*	0.1
	MIP1β	114 *(77, 152)*	136 *(105, 158)*	0.2
Th17	IL17A	2.64 *(1.60, 3.04)*	1.72 *(1.20, 2.39)*	0.018^*^
	IL17AF	0 *(0, 0.1)*	0.001 *(0, 0.3)*	0.6
	IL17B	0.31 *(0.14, 0.44)*	0.67 *(0.31, 0.97)*	0.016^*^
	IL17C	0 *(0, 0.1)*	0.00 *(0.00, 0.00)*	>0.9
Vascular Injury	CRP	2,162,710 *(862,164, 7,718,677)*	520,619 *(284,546, 1,538,951)*	0.014^*^
	ICAM1	68,440 *(463,980, 698,292)*	391,945 *(323,475, 593,272)*	0.004^*^
	VCAM1	612,265 *(540,375, 752,649)*	425,374 *(355,599, 578,439)*	<0.001^*^
	SAA	1,928,395 *(1,323,845, 5,521,066)*	1,279,690 *(751,070, 2,022,183)*	0.045^*^
Angiogenesis	Flt1	118 *(111, 145)*	142 *(128, 180)*	0.022^*^
	VEGF	343 *(139, 377)*	139 *(82, 202)*	0.013^*^
	VEGF-C	529 *(486, 578)*	533 *(496, 690)*	0.4
Growth Factors	TSLP	0	0	0.4
	IL7	13.7 *(9.9, 18.0)*	15.1 *(11.2, 18.6)*	0.7

^1^Factors grouped based on their biological functions. ^2^Data expressed as pg/ml serum cytokine concentration; ^3^p-value from Wilcoxon signed rank test. Asterisk (^*^) indicates statistical significance.

### 3.3 Identification of factors indicative of SSTI cases

**Figure 2:**
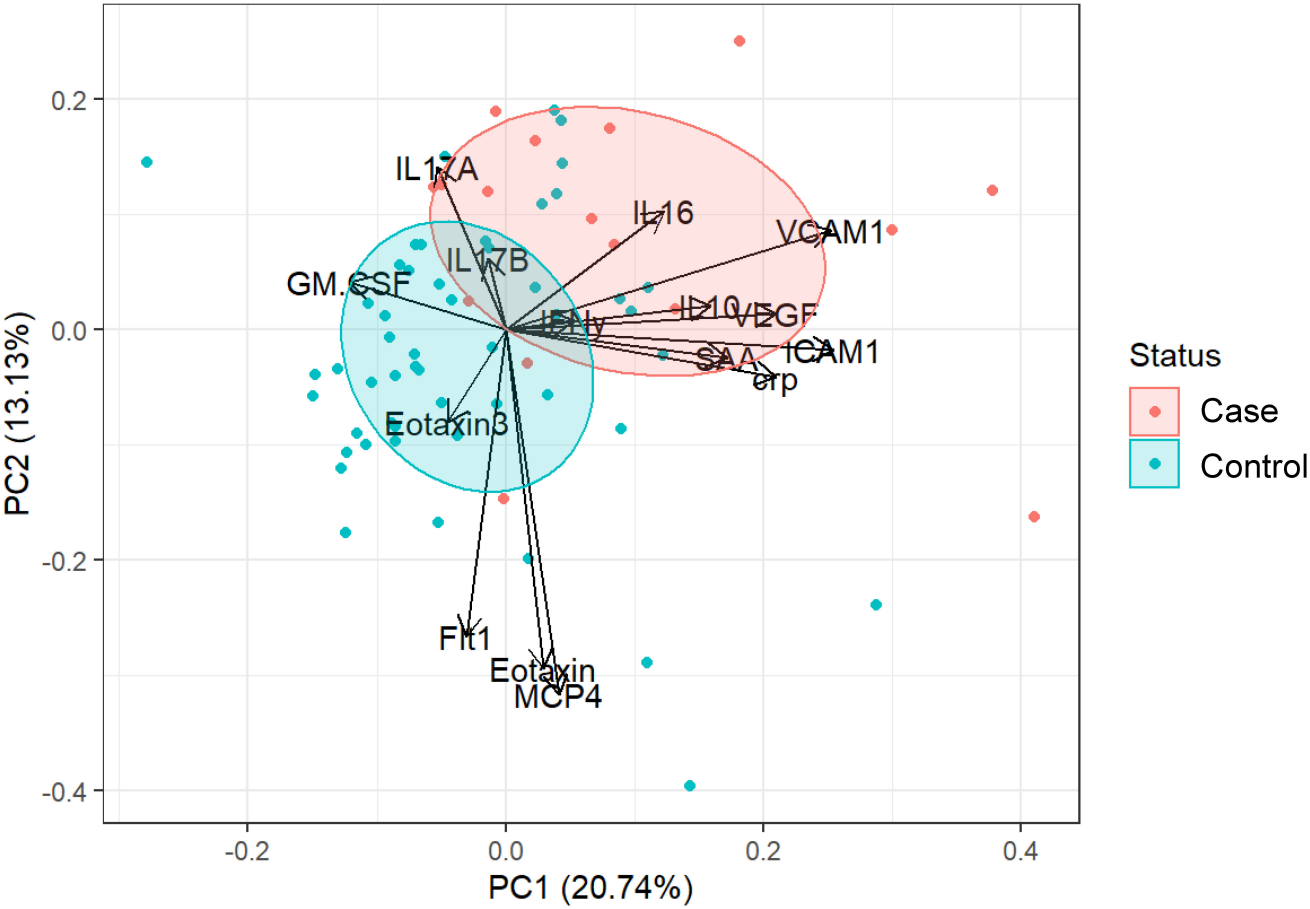
Combining all significant factors to establish cohort-specific cytokine signatures. PCA plot of cytokine responses significantly (p ≤ 0.1) different between controls (blue) and cases (red). Loading vectors showing the direction of the contribution of each parameter to the PCA. Ellipses correspond to 50% confidence intervals for each group.

All significantly different serum factors (p ≤ 0.1) were used as input for a principal component analysis (PCA) (**Figure 2**). The objective was to determine whether serum factor profiles of SSTI cases vs. control participants are sufficiently distinct from each other. The analysis also determined which of the factors contributed to a cohort-specific profile. The results showed that IL17A, IL16, IL10, and factors associated with vascular injury (VCAM1, ICAM1, SAA, CRP) and angiogenesis (VEGF) were prevalent in the profile of SSTI cases. The profile of controls was dominated by chemokines (Eotaxin-3, Eotaxin, MCP4), pro-inflammatory cytokines (GM-CSF), and the angiogenetic factor Flt1. The area of the ellipses for each cohort revealed little overlap between cases and healthy individuals.

**Figure 3:**
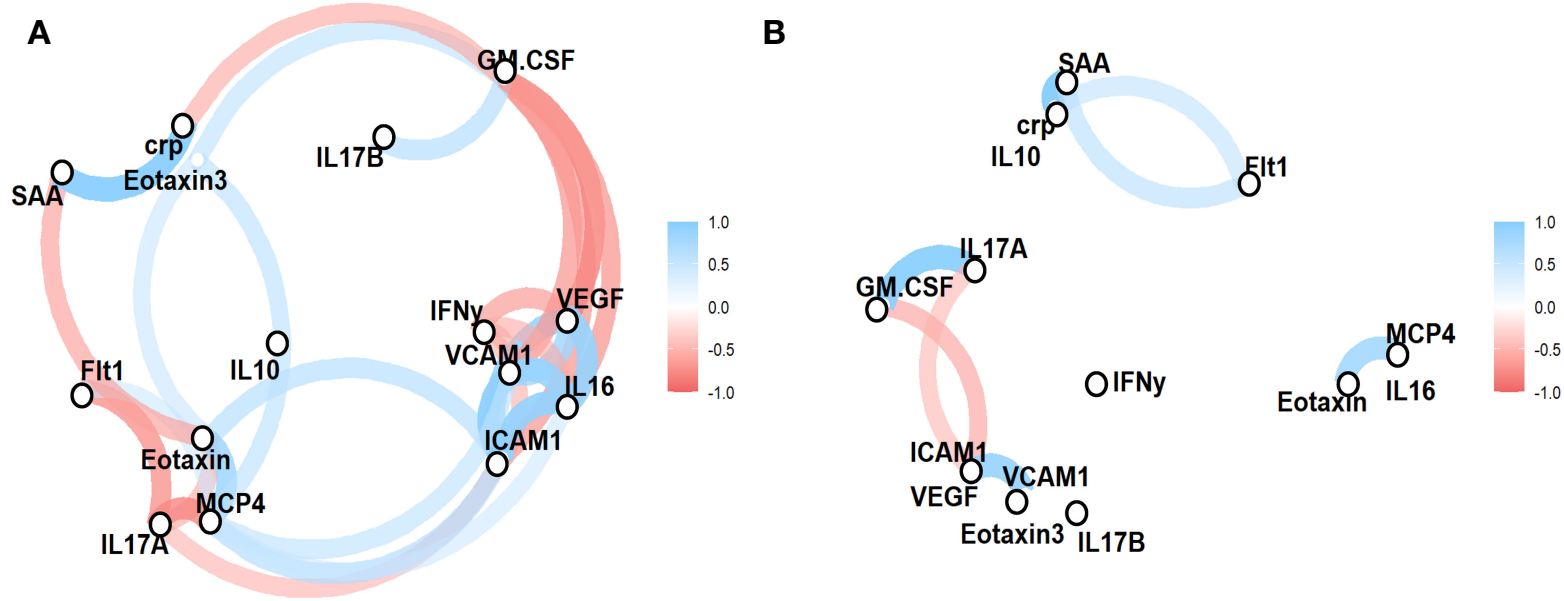
Functional relationships between serum factors reveal cohort-specific serum factor profiles. Concentrations of serum factors significantly different in SSTIs **(A)** and healthy controls **(B)** were used to established correlation networks. The color and size of the network lines are corresponding to pairwise Pearson correlation coefficients (only correlations ≥ 0.5 are shown).

Lastly, we sought to determine the functional relationship between the factors that had been identified to contribute to the cohort-specific serum factor profile of -SSTI cases vs. healthy controls. Correlation networks were generated for both cohorts to investigate the functional interplay between serum factors significantly different between cases and controls (**Figure 3**). In case of control individuals, positive correlations between factors associated with vascular injury (SAA, CRP) and Flt1, between GM-CSF and IL17A, and the chemokines Eotaxin and MCP4 were observed. A strong negative correlation was observed between ICAM1, GM-CSF and IL17A.

In individuals with SSTIs, there were a wide range of correlations between vascular injury (SAA vs. CRP, ICAM1 vs VCAM1), angiogenesis factors (Flt1 vs IL17A and Eotaxin, VGEF vs IFNγ), and chemokines (Eotaxin vs. MCP4), proinflammatory GM-CSF vs IL17B and IFNγ vs VCAM1.

## 4 Discussion

Housing situations where people live in close contact are considered a perfect environment for propagation of infectious diseases. Such settings are often unavoidable as they may be part of leisurely activities (e.g., cruise ships), schooling (e.g., dormitories), the work environment, or training settings. These settings offer a unique opportunity for studying the dynamic and kinetic of a variety of infectious agents and identify biomarkers of exposure and various stages of infections. For example, the crowded living conditions of the highly regimented military training creates an environment associated with a high risk for trainees of acquiring *S. aureus* colonization and developing SSTIs.

We utilized the samples of an observational, longitudinal cohort study of SSTI for an exploratory investigation to determine the impact of impending SSTIs on the profile of serum factors. The MSD multiplex testing platform utilized in this study was invaluable for this exploratory study as the wide linear range of the quantitation and the high reproducibility was essential to enabling the analyses with a small sample volume (14, 16). Other testing platforms require serial dilutions of the test samples to ensure that the concentration of the respective factor falls within the linear range thus enabling quantitation. Testing in technical replicates is recommended due to the variability in the sample acquisition (bead-based flow cytometry) or variability in timing and pipetting accuracy (ELISA) further increasing the requirement of a large sample volume. The applied MSD platform, however, enabled the quantification of all 54 factors based on a single dilution of the serum sample. This, in turn, opens opportunity for other immunoprofiling studies including pediatric or critically ill patients to identify biomarkers of infection and/or disease.

In our study, the distribution of SSTI occurrence appeared bimodal (**Figure 1**) with a peak of SSTIs within the first month and towards the end of the training. Moving into group housing and increased contact among the trainees resulted in colonization of individuals and the first wave of SSTIs. The second peak may be due to chronic stress caused by the continued training and need for top performance. Stress is immunosuppressive over extended period of time and increases susceptibility (17–20). The impact of short-term vs. chronic stress has even been reported to promote the emergence of malignancies such as skin cancer (21). Immune cells have hormone receptors and corticosteroids influence immune functions [reviewed in (22)]. The possibility that increased susceptibility to SSTI is caused by concurrent other infections cannot be excluded as the participants were not screened for additional pathogens.

Our investigation yielded several novel findings. In particular, we identified candidate biomarkers of pre-illness states of *S. aureus* soft tissue infections. The serum factor profiles of pre-illness were marked by increases in the concentration of factors associated with inflammation (IFNγ, IL10, IL17A), tissue damage (CRP, ICAM1, VCAM1, SAA), and would healing (Flt1, VGEF). This serum factor profile associated with infection demonstrates a highly pro-inflammatory response. Apart from the classic inflammatory cytokines IFNγ, IL10, IL17, early acute phase proteins (CRP (23), SAA (24) also contributed to inflammation and onset of immune responses. The soluble receptors ICAM1, VCAM1 have shown their potential as biomarkers [prognostic, diagnostic (25)] for inflammatory responses in a variety of diseases. VEGF, an angiogenetic factor, has been reported to play a crucial role in bacterial pathogen-induced inflammatory responses (26) including in the immune defense against *Streptococcus pyogenes* (27). Also noteworthy is the fact that *S. aureus* triggers VEGF in mast cells suggesting that the increased presence of VEGF in the blood could serve as a marker for bacterial infections (28).

The correlation networks (**Figure 3**) visualized the functional interplay between serum factors and distinct “profiles” between participants with SSTI vs. control subjects. The correlation networks of individuals with SSTIs revealed a wide range of interactions between serum factors and reflected the various layers of innate and adaptive immune responses and the induction of factors associated with wounds and wound healing.

In addition, the present study implicated Flt1, the soluble VEGF-R1 receptor with anti-angiogenetic functions (29), being involved in immune responses against pathogens. To date, this factor has been shown to play a major role in complications associated with pregnancy (30) and potential function as marker for the severity of sepsis (31).

There is a substantial knowledge gap for early markers of infections. For example, Dengue-infected individuals often present only after peak viremia which leaves supportive therapy as the only treatment option (32). This emphasizes the critical need for biomarker discovery as identifying early stages of an infection or disease would increase the prognostic outcome. The challenge with SSTI is that colonization may not be an accurate predictor of subsequent infections (33). A meta-analysis of 29 studies investigated results from exoproteomics, serological responses, serum cytokines in the serum of individuals with *S. aureus* SSTIs, bacteremia and subclinical colonization in search of predictive factors for SSTIs (33). The factors highlighted in this meta-analysis were IFNγ, IL2, IL10, IL4, IL6, IL8, IL17A, TNFα, IP10, MCP1, MIG, and RANTES. In agreement with our results, the meta-analysis reported significantly increased levels of IFNγ, IL2, IL6, IL8, IL10, IL17A. However, we did not observe significant increase in TNFα, or MCP in sera of patients with SSTI infections. The serum factors IL6, IL8, and IL17 mediate neutrophil recruitment, a hallmark of disseminated bacterial infections (34–36). Activated neutrophils produce a range of soluble factors that further amplify the immune response. These factors include IP10 and MIG, which have been shown to recruit T cells to sites of inflammation (37).

A study investigating the diagnostic value of laboratory parameters (blood levels of CRP and leukocytes) revealed the potential of CRP to discriminate between bacterial SSTIs and herpes zoster (38) supporting our findings.

For our study, we selected samples from individuals in the pre-illness state of a SSTI and compared them to baseline data from individuals without SSTIs throughout the observation period. While this impacted the sample size of our study, it offered the opportunity to identify candidate biomarkers. The other limitation of our study was the fact that all study participants were male and Caucasian. The promising results from this exploratory profiling will lay the foundation for a detailed assessment of changes in the serum factor profiles throughout different stages of the infection and consider sex and race as additional factors that may influence the serum factor profiles.

In conclusion, the results demonstrated changes in the serum levels of cytokines/chemokines that can be associated with SSTI cases thus paving the way to explore these factors as biomarkers for susceptibility to SSTIs.

## Data availability statement

The original contributions presented in the study are included in the article/supplementary material. Further inquiries can be directed to the corresponding authors.

## Ethics statement

The studies involving humans were approved by Institutional review board of the Uniformed Services University of Health Sciences, Bethesda, MD. The studies were conducted in accordance with the local legislation and institutional requirements. The participants provided their written informed consent to participate in this study.

## Author contributions

EB-L: Conceptualization, Formal analysis, Software, Writing – original draft. EM: Conceptualization, Data curation, Funding acquisition, Writing – original draft. ED: Methodology, Writing – review & editing. DT: Funding acquisition, Resources, Writing – review & editing. PC: Investigation, Resources, Writing – review & editing. ME: Resources, Writing – review & editing. KM: Funding acquisition, Resources, Writing – review & editing. JB: Funding acquisition, Project administration, Resources, Writing – review & editing. SC: Formal analysis, Investigation, Software, Writing – review & editing.

## Author disclaimer

Material has been reviewed by the Walter Reed Army Institute of Research. There is no objection to its presentation and/or publication. This paper has been approved for public release with unlimited distribution. The investigators have adhered to the policies for protection of human subjects as prescribed in AR 70–25. EB-L, EHD, DRT, PMC, JWB, SC are government employees and this work was prepared as part of their official duties. Title 17 U.S.C. § 105 provides that copyright protection under this title is not available for any work of the U.S. Government. The views expressed in this paper are those of the authors and do not necessarily represent the views of the Henry M. Jackson Foundation for the Advancement of Military Medicine, Inc., National Institute of Health or Department of Health and Human Services, the Uniformed Services University of the Health Sciences, Brooke Army Medical Center, Defense Health Agency, the Departments of the Army, Navy, and Air Force, Department of Defense (DoD), nor the U.S. Government.
